# A mid year comparison study of career satisfaction and emotional states between residents and faculty at one academic medical center

**DOI:** 10.1186/1472-6920-6-36

**Published:** 2006-07-07

**Authors:** Donald E Girard, Dongseok Choi, Jamie Dickey, Kristen Wessel, Donald Austin

**Affiliations:** 1 Graduate Medical Education, School of Medicine, Oregon Health & Science University, Portland, OR, USA; 2 Department ofPublic Health and Preventive Medicine, School of Medicine, Oregon Health & Science University, Portland, OR, USA; 3 Department of Psychiatry, School of Medicine, Oregon Health & Science University, Portland, OR, USA; 4 Department of Anesthesiology, School of Medicine, Oregon Health & Science University, Portland, OR, USA

## Abstract

**Background:**

The Accreditation Council for Graduate Medical Education's (ACGME) new requirements raise multiple challenges for academic medical centers. We sought to evaluate career satisfaction, emotional states, positive and negative experiences, work hours and sleep among residents and faculty simultaneously in one academic medical center after implementation of the ACGME duty hour requirements.

**Methods:**

Residents and faculty (1330) in the academic health center were asked to participate in a confidential survey; 72% of the residents and 66% of the faculty completed the survey.

**Results:**

Compared to residents, faculty had higher levels of satisfaction with career choice, competence, importance and usefulness; lower levels of anxiousness and depression. The most positive experiences for both groups corresponded to strong interpersonal relationships and educational value; most negative experiences to poor interpersonal relationships and issues perceived outside of the physician's control.

Approximately 13% of the residents and 14% of the faculty were out of compliance with duty hour requirements. Nearly 5% of faculty reported working more than 100 hours per week. For faculty who worked 24 hour shifts, nearly 60% were out of compliance with the duty-hour requirements.

**Conclusion:**

Reasons for increased satisfaction with career choice, positive emotional states and experiences for faculty compared to residents are unexplained. Earlier studies from this institution identified similar positive findings among advanced residents compared to more junior residents. Faculty are more frequently at risk for duty-hour violations. If patient safety is of prime importance, faculty, in particular, should be compliant with the duty hour requirements. Perhaps the ACGME should contain faculty work hours as part of its regulatory function.

## Background

The Accreditation Council for Graduate Medical Education's (ACGME) new requirements limiting work hours, requiring demonstration of competence in six core areas, and emphasizing the importance of a humane, collegial environment, raise multiple challenges for academic medical centers; i.e., for example new resources to care for patients whose care cannot be provided by residents, new curricula to be learned, taught, and methods developed for evaluation are just two. From recently published reports, we know more about the course of emotion and attitude change during training and differences among resident groups. We have been provided information about resident "burnout", physician wellness, and the importance of self-reflection [1-8]. Our own studies have shed light on both residents' attitudes and emotions as well as perceived positive and negative aspects of training – across specialties, and over training years [9-13]. Finally there are some recent reports about change in satisfaction and work hours among small groups of faculty and residents after the implementation of the ACGME requirements [14-18]. However, no previous study has undertaken an evaluation of career satisfaction, emotional states, perceived positive and negative experiences, work hours and sleep among all faculty and residents in an academic institution simultaneously. While other studies have looked at single specialties, this research addresses these issues for all specialties in the academic medical center.

This study utilized a previously validated survey instrument (Profile of Mood States[13]) to evaluate satisfaction with career choice and emotional states among all residents and all faculty in one large academic medical center. The survey took place at mid-year, a time that typically represents the peak period of negative feelings and attitudes among residents about their career choices [10].

## Methods

Six hundred and twenty-five residents, representing 58 ACGME accredited residency programs, and 705 faculty, representing all clinical faculty at OHSU who utilized the electronic data management system were surveyed between January and February 2005 using a previously validated instrument to evaluate levels of satisfaction with career choice, emotional states, factors that were particularly satisfying or dissatisfying about the professional experience, hours worked and slept [13]. There were 282 faculty, mostly community based physicians, who were excluded from the survey. While they participate in resident teaching, they are not part of the core-faculty. All responses were anonymous and confidential. The survey, divided into six sections, asked residents and faculty to:

• Rate levels of satisfaction with career choice by choosing one of five alternative statements ranging from "I regret the decision and may drop out" (one on a five point scale) to "I am consistently pleased with my decision" (five point).

• Indicate agreement with statements (33 for residents and 32 for faculty) describing positive or negative aspects of the professional experience.

• Rate a list of 19 adjectives, each describing a positive or a negative emotion or belief on a five point Likert scale.

• Rate "real" stress level compared to what was previously expected.

• Provide narrative comments about positive and negative aspects of the professional experience.

• Report duty hours and sleep, averaged over the previous 4 weeks.

The original survey instrument, designed for residents, was modified slightly to assure that the questions were pertinent to the faculty cohort. The data were first explored by using descriptive statistics for both resident and faculty cohorts. For further analyses, participants were divided into residents and faculty groups. Two sample t-tests and χ^2 ^tests were employed to evaluate differences in the emotional states between the two groups.

## Results

The demographic distributions (age, gender, race) for the resident respondents are very similar to those of all residents (data not shown) and are similar to those for the graduates of US medical schools[19] The demographic distributions for the entire faculty from an institutional database are also representative of faculty in US medical schools [20] although faculty demographic information was not collected with the survey.

Table 1 shows the means and sample standard deviations for answers to questions relating to career satisfaction, emotional states and perceived levels of job stress. The data are not specified for specialty. While both groups were generally satisfied, the faculty were significantly more satisfied with career choice and also felt more stress than expected compared to the residents. The overall average score of career choice satisfaction for residents was 3.98 out of 5 with a 0.88 sample standard deviation. For the faculty scores were 4.29 out of 5, with a .81 sample standard deviation, significantly higher for the faculty than for the residents (p-value = 0.0%). The average scores of stress level for the faculty were 3.15 out of 5 with a 0.84 sample standard deviation while 3 out 5 with a 0.90 sample standard deviation for residents.

For the questions related to emotional states, the faculty physicians were more positive and less negative than the residents for the majority of choices in all applicable categories. The faculty were significantly more positive and less negative compared to the residents for 8 out of 9 positive emotional descriptors and 9 out of 10 negative emotional descriptors, respectively. Interestingly, there were no differences in "Relieved" and "Angry" between residents and faculty. It is worth noting that the directions of the differences in satisfaction and emotional states were consistent.

**Figure 1:**
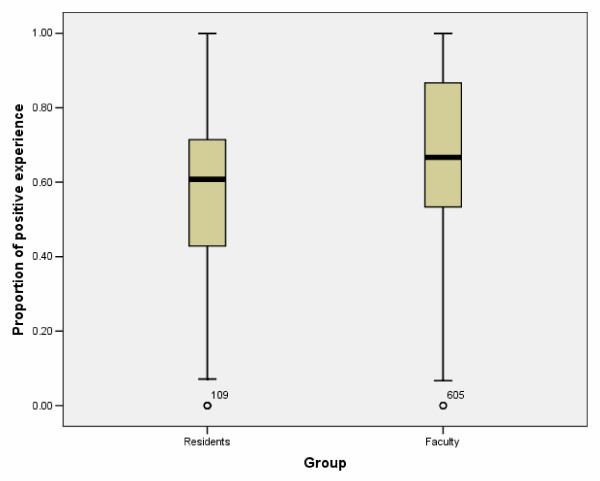
Boxplots of the proportions of positive experiences by residents and faculty.

**Figure 2:**
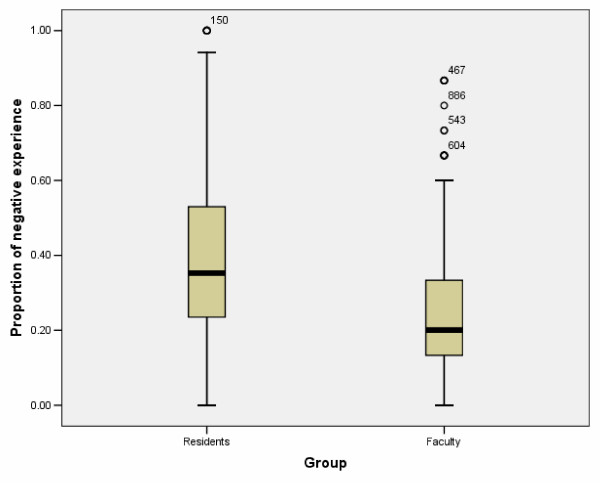
Boxplots of the proportions of negative experiences by residents and faculty.

In the survey, there were two separate groups of questions corresponding to positive and negative aspects of the professional experience, respectively. The proportion of positive experiences for a participant was computed by taking the ratio of the number of "yes" responses to the total number of positive questions and in similar fashion for negative experiences. The average proportions of positive and negative aspects of the professional experience also differed significantly between faculty and residents. Figures 1 and 2 display the distributions of proportions of positive experiences and negative experiences grouped for faculty and residents respectively. The proportions of positive experiences were higher (68% vs. 58%, p-value = 0.0%) and proportions of negative experiences lower (23% vs. 39%, p-value = 0.0%) for the faculty compared to the residents.

**Figure 3:**
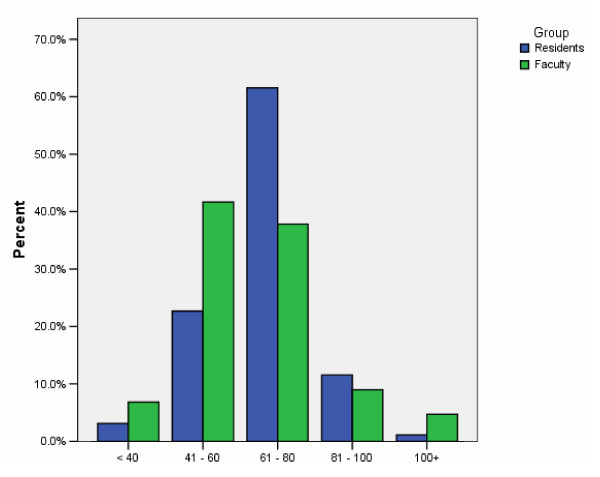
Self-reported average working hours over 4 weeks averaged over 4 weeks.

**Figure 4:**
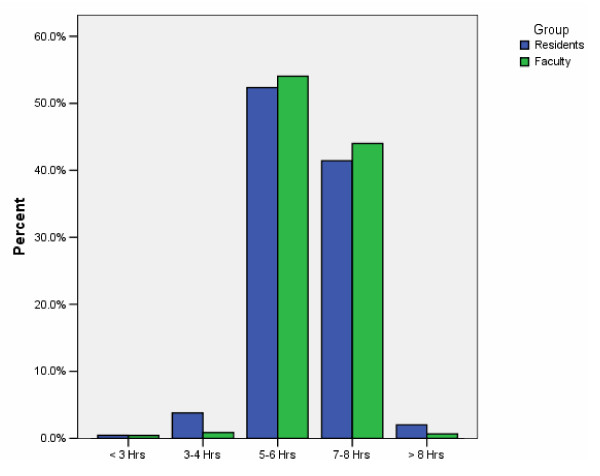
Self-reported sleep hours averaged over one week.

Figures 3, 4 provide data on work hours and sleep for the two groups. Thirteen percent of the residents and 14% of the faculty reported working more than 80 hours per week (Figure 3). In addition, 2% of the residents but 9% of the faculty reported not having one day off for every 7 working days. Fourteen percent of the residents but 60% of the faculty who had duty hour assignments of 24 hours did not leave the hospital within 6 hours after the end of the 24-hour period. Figure 4 shows the distributions of self-reported sleep hours averaged over one week. About 4.2% of residents reported less than 4 hours sleep while 1.3% faculty reported similarly. There were statistical differences with a p-value = 1.5% by χ^2 ^test in the distributions of sleep hours between the groups although the average sleep hours were comparable between them.

Ninety-nine percent of the residents and faculty respondents provided narrative comments about factors that positively influenced their experiences and 81% provided narrative comments about negative factors influencing their experiences. Virtually all the positive and negative themes were identified in the 33 statements presented on the survey instrument.

## Discussion

No previous study has compared markers of career satisfaction, emotional states, opinions about positive and negative aspects of the "physician experience", compliance with the ACGME duty hour requirements and sleep attainment in both resident physicians and their faculty simultaneously. The survey has been validated to reflect accurately residents' emotions and attitudes about career satisfaction. Although it has not been validated to reflect faculty opinions in these categories, there is little reason to suspect that the instrument would not be applicable to them. The response rates were excellent – nearly 72 percent of residents and 66 percent of faculty. The reliability of the instrument is supported by the fact that all of the narrative comments were consistent with at least one of the 33 choices about positive and negative experiences provided in the survey questions.

The study was intentionally implemented at mid-year, which is the most negative time for residents' in their training experiences. Accordingly, beliefs about career satisfaction, emotional states and about their professional experiences will typically be at their low points at this time. It is thus reassuring that satisfaction with career choice and emotional states were actually positive for both residents and faculty groups, an indication that the professional milieu is positive. The reasons faculty were more positive and less negative than the residents in career satisfaction, emotional states and positive and negative perceptions of the experience, and at the same time more stressed about their jobs are not understood. Obviously more questions have been raised than answered and these data raise abundant opportunities for more research. However, in previous studies published from this institution, we have consistently noted that residents have more positive emotions and attitudes about their professional experiences as they advance in training [9-11]. They recognize their competence and importance, and their anxiety and depression dissipate. Thus, we believe that faculty's more positive attitudes and emotions reflect the changes in the continuum of advancement observed during residency. These findings are also of particular interest because both groups completed the survey after implementation of the ACGME duty hour requirements. There are a number of recent reports that focus on satisfaction and work hours for residents and faculty, and in some, before and after implementation of the duty hour requirements. Unfortunately all focused on single specialties, had small sample sizes, used variable methodologies, and their findings are not consistent [16-18].

One might consider any number of specific changes that make the faculty more positive, divided for convenience into professional and personal characteristics. Professional ones likely include successful completion of training, assumption of supervisory and teaching roles, as well as academic job stability and security. Personal characteristics likely include age, established relationships and clear support systems, improved financial stability, time for other interests, improved efficiencies. Of perhaps these differences amount simply to "generational gaps".

One unanticipated finding of the study relates to reported work hours and sleep for the two groups. It is interesting that the average sleep hours were surprisingly similar for the cohorts. Thus good or bad sleep patterns seem to be shared by both groups, and residents seem to have at least the same opportunities for sleep as do their faculty. While the vast majority of respondents in both groups were nearly compliant with the ACGME duty hour requirements, it is a surprise that the faculty are slightly less compliant than the residents. And, while more residents than faculty reported working up to the maximum allowable 80 hours per week, more than 60% of the faculty who had duty hour assignments of 24 stayed more than 30 hours and did not have 10 hours between shifts. The work hour data for the residents support the fact that the institution is invested in assuring that the ACGME requirements are met. However, the same cannot be said about a commitment to the faculty. The faculty's work hour data raise significant concern about their excesses and thus patients' safety. While these numbers are not large, and represent a relatively small number of faculty, these individuals are likely in units where there are critically ill patients. Neurobiologists have warned institutional and academic leaders that sleep deprivation and its consequences are particularly difficult for older individuals. Sleep deprivation in older individuals may have more significant implications for patient safety than the same degree of sleep deprivation for younger individuals. If this is true, perhaps the ACGME work hour requirements should be applied to faculty who work in its accredited programs.

## Conclusion

• Compared to the residents, faculty were notably more positive about career satisfaction, emotional states, and opinions about the professional experience in one large institution. Reasons for differences are unknown but likely relate to the same evolution seen among residents between the first and senior years of training as they recognize their competence and importance.

• While the residents' work hours are generally compliant with the ACGME requirements, those of a significant cohort of faculty are working more than 80 hours per week. And these faculty are likely caring for critically ill patients. This raises significant concern about patient safety and should be addressed by the appropriate oversight agencies.

## Competing interests

The author(s) declare that they have no competing interests.

## Authors' contributions

DEG supervised the whole project, designed the survey and drafted the manuscript; DC performed statistical analyses, interpreted the results and drafted the manuscript; JD designed the survey and revised the draft critically, KW revised the survey questionnaire, revised the draft critically; DA designed the survey questionnaire related to stress and work experience.

## Pre-publication history

The pre-publication history for this paper can be accessed here:



## Figures and Tables

**Table 1:** The mean scores of career satisfaction, stress and emotional states.

	Residents		Faculty		
	Mean	S D	Mean	S D	p-value

Satisfaction with career choice	3.98	0.88	4.29	0.81	0%
More stress than expected	3.00	0.90	3.15	0.84	4%
Appreciated	3.08	0.96	3.61	0.98	0%
Com petent	3.39	0.80	4.13	0.76	0%
Energized	2.75	0.96	3.20	0.94	0%
Excited	3.26	0.96	3.45	0.95	2%
Im portant	2.88	0.91	3.25	0.95	0%
Relieved	2.66	0.98	2.53	1.02	24%
Respected	3.14	0.89	3.79	0.83	0%
Useful	3.55	0.87	4.15	0.82	0%
Valued	3.14	0.94	3.65	0.96	0%
Angry	2.10	1.11	1.92	1.02	14%
Anxious	2.85	1.13	2.29	1.10	0%
Bored	2.03	0.97	1.78	0.95	0%
Depressed	2.39	1.18	1.83	0.97	0%
Defeated	2.06	1.14	1.70	0.96	0%
Fatigued	3.40	1.12	3.04	1.19	0%
Inferior	2.31	1.14	1.57	0.84	0%
Lonely	2.26	1.22	1.67	0.98	0%
Nervous	2.81	1.05	2.19	1.02	0%
O verwhelm ed	2.96	1.08	2.91	1.18	5%

• *SD: sample standard deviation*

• P-values were from χ^2 ^test between residents and faculty on a five-point Likert scale (1-do not feel this way at all, and 5-feel strongly this way).
